# Cardiac symptoms in patients 3–6 months after contracting COVID-19– data from the polish STOP-COVID registry

**DOI:** 10.1186/s12879-025-10774-0

**Published:** 2025-04-09

**Authors:** Mateusz Babicki, Joanna Kapusta, Damian Kołat, Żaneta Kałuzińska-Kołat, Agnieszka Mastalerz-Migas, Piotr Jankowski, Michał Chudzik

**Affiliations:** 1 Department of Family Medicine, Wroclaw Medical University, Wroclaw, 51-141 Poland; 2 Department of Internal Diseases, Rehabilitation and Physical Medicine, Medical University of Lodz, Lodz, 90-647 Poland; 3 Department of Biomedicine and Experimental Surgery, Medical University of Lodz, Narutowicza 60, Lodz, 90-136 Poland; 4 Department of Functional Genomics, Medical University of Lodz, Żeligowskiego 7/9, Lodz, 90-752 Poland; 5 Department of Internal Medicine and Geriatric Cardiology, Medical Centre for Postgraduate Education, Warsaw, 01-813 Poland; 6 Department of Nephrology, Hypertension and Family Medicine, Medical University of Lodz, Lodz, 90-549 Poland

**Keywords:** Long COVID, Cardiovascular disease, COVID-19, SARS-CoV-2, Omicron

## Abstract

**Background:**

Common complaints of long COVID patients are cardiac symptoms such as fatigue, weakness, and a feeling of palpitations. The study aimed to investigate the clinical features of patients with persistent cardiological symptoms occurring within 3 to 6 months after COVID-19. Differences in ambulatory blood pressure monitoring (ABPM), Holter ECG (electrocardiogram) and Echocardiography between people with and without persistent cardiological symptoms were evaluated. We also assessed whether the symptoms of anxiety and depression may be implicated in the clinical outcomes.

**Materials and methods:**

This was a retrospective study of patients affiliated with the STOP-COVID registry who attended a follow-up visit 3–6 months after undergoing COVID-19. The visit assessed the clinical symptoms present and performed tests: ABPM, Holter ECG and Echocardiography. 504 patients additionally had GAD-2 (Generalized Anxiety Disorder 2-item) and PHQ-2 (Patient Health Questionnaire-2) tests performed.

**Results:**

The analysis included 1080 patients. At least 1 of the analyzed symptoms was present in 586 patients (54.3%). The most common symptom was fatigue (38.9%). Comparing patients with or without palpitations showed that the mean value of ventricular extrasystole was higher in the former group (*p* = 0.011). Comparing patients with and without cardiac symptoms, there were differences in the mean values of the PHQ-2 (*p* = 0.022) and GAD-2 (*p* < 0.001) scales, as well as in the percentage of responses related to the risk of anxiety or depression.

**Conclusion:**

Cardiological symptoms are common among health issues that patients must face after contracting COVID-19. People with palpitations had more excessive ventricular extrasystoles than patients without these symptoms.

**Trial registration:**

Our retrospective study was based on analysis of medical data of patients with COVID-19 treated on out-patient basis in the STOP-COVID registry of the Polish Long-Covid Cardiovascular (PoLoCOV-CVD) study (ClinicalTrials.gov identifier– NCT05018052, the registration date 29.05.2020). Consent to conduct the study was obtained from the Bioethics Committee of the District Medical Chamber in Lodz (no. KB-0115/2021).

## Background

The Coronavirus Disease 2019 (COVID-19) affected over 774 million individuals and caused more than seven million deaths globally [1]. In Poland, 7.7 million cases and 120,000 deaths due to SARS-CoV-2 (severe acute respiratory syndrome coronavirus 2) were recorded by May 2024 [2]. Even though the World Health Organization (WHO) announced the end of the global public health threat in 2023, SARS-CoV-2 still poses a risk, mainly due to persistent symptoms after COVID-19, collectively termed Long COVID (LC) [3, 4]. This term was defined by WHO to describe symptoms that persist or appear after the acute phase of SARS-CoV-2 infection and extend beyond the initial four-week period. It is differentiated into “persistent symptomatic COVID-19” (symptoms persisting for 4 to 12 weeks) and “post-COVID-19 syndrome” (persistence of symptoms for more than 12 weeks) [5, 6]. Its incidence varies depending on the data source: it is estimated at approximately 10% of non-hospitalized people [7, 8]. The long-term complications of COVID-19 reported by patients are very diverse, affecting the respiratory, nervous, digestive, and cardiovascular systems [9, 10]. LC is characterized by persistent symptoms such as fatigue, muscle weakness, shortness of breath, heart rhythm disturbances, arthralgia, and neurological complications such as impaired memory, “brain fog”, and headaches [11, 12]. Some of the most common manifestations of Long COVID include cardiac symptoms [13, 14]. Long COVID poses a significant economic burden and worsens the quality of life [15].

The course of SARS-CoV-2 infection and the possible occurrence of long-term consequences are influenced by many factors. They include age, gender, immune response, and chronic diseases (e.g., diabetes and respiratory or cardiovascular diseases), but also the virus variant [16–18]. Since 2022, the most prevalent variant in Poland was Omicron [19]. Its greater infectivity, shorter incubation period, and higher replication efficiency result in a milder disease course, which makes Omicron completely different compared to the previous variants, i.e., Alpha or Delta [17, 20, 21]. It has been shown that Omicron contributes less to the development of long-term complications of COVID-19. This is confirmed by large studies in many countries around the world [22]. The reasons why the Omicron variant has a lower risk of developing complications are not known. The reduced long COVID risk after Omicron infections could result from the intrinsic properties of SARS-CoV-2 variants to cause long-term health problems, as well as differences in vaccination and population immunity to SARS-CoV-2 [23].

A literature review published by Tsampasian et al. showed that one of the most likely mechanisms leading to the development of Long COVID and cardiac symptoms is an elevated immune response during the acute phase of COVID-19. Persistently elevated levels of pro-inflammatory cytokines and lymphocytes B and T were noted [24]. Currently, there is no evidence of the influence of genetic predisposition on the risk of developing Long COVID, but related research is ongoing [25]. Moreover, attention is currently being paid to the influence of the mental condition on the symptoms of Long COVID [26, 27]. Previous studies have shown that people with long COVID syndrome are more likely to develop depression, anxiety and sleep disorders, or even post-traumatic stress disorder. It is believed that the basis for their coexistence may be due to the persistence of clinical symptoms, which can affect quality of life and carry both social and economic consequences. On the other hand, it is believed that mechanisms from the immune system may also play a role [28]. Other studies suggest that depression and anxiety are comorbidities and predictors of long COVID [29].

In order to unequivocally identify a cardiovascular disorder, it is necessary to perform certain additional tests, which may include laboratory measurements, blood pressure measurements, ECG, as well as imaging studies such as echocardiography [30]. Previous studies conducted on patients after COVID-19 have shown that echocardiographic abnormalities such as left ventricular diastolic dysfunction and right ventricular dysfunction were common in patients with persistent long COVID [31–33]. Moreover, assessment of ejection fraction allows the diagnosis of heart failure. In the assessment of myocardial damage, ECG evaluation can also be a useful parameter, which has been confirmed in other studies, including those evaluating predictors of long COVID [34–36].

In light of the above data, there is a need for studies assessing the association of SARS-CoV-2 infection with cardiovascular disorders. Therefore, this study aimed to investigate the clinical features of patients with persistent cardiological symptoms occurring within 3 to 6 months after COVID-19. Differences in ambulatory blood pressure monitoring (ABPM), Holter ECG and Echocardiography between people with and without persistent cardiological symptoms were evaluated. In addition, we assessed whether the symptoms of anxiety and depression may be implicated in the clinical outcomes.

## Materials and methods

### Study group characteristics

This is a retrospective study on a group of patients associated with the STOP-COVID project (ClinicalTrials.gov ID: NCT05018052). Consent to conduct the study was obtained from the Bioethics Committee of the District Medical Chamber in Lodz (no. KB-0115/2021).

Of the 6100 patients reviewed as part of the STOP COVID registry, 1080 met the criteria and were included in further analyses. Before participating in the study, patients were informed about the methodology and study goals, which allowed them to give their informed consent.

Patients were divided into two groups depending on the occurrence of cardiac symptoms after COVID-19: group A– patients with cardio Long COVID (cardio LC = 586) and group B– patients without cardio Long COVID (non Cardio-LC = 494). According to the adopted definition, Long COVID was diagnosed in patients who had at least 1 symptom 3 months after suffering from COVID-19, which could not be explained by other causes. But only symptoms suggesting cardiac problems were analyzed, such as excessive fatigue, shortness of breath, chest pain, fainting and the feeling of heart palpitations (group A). The second analyzed group consisted of patients who had none of the above-mentioned cardiological symptoms (group B). Additionally, patients were divided according to the period of the disease, taking into account the period before and during the dominance of the Omicron variant. The cut-off date was January 2022, in accordance with a previous publication indicating that the Omicron variant was responsible for > 95% of the causes of infection in Poland [19].

### The inclusion criteria for this research

**Fig. 1:**
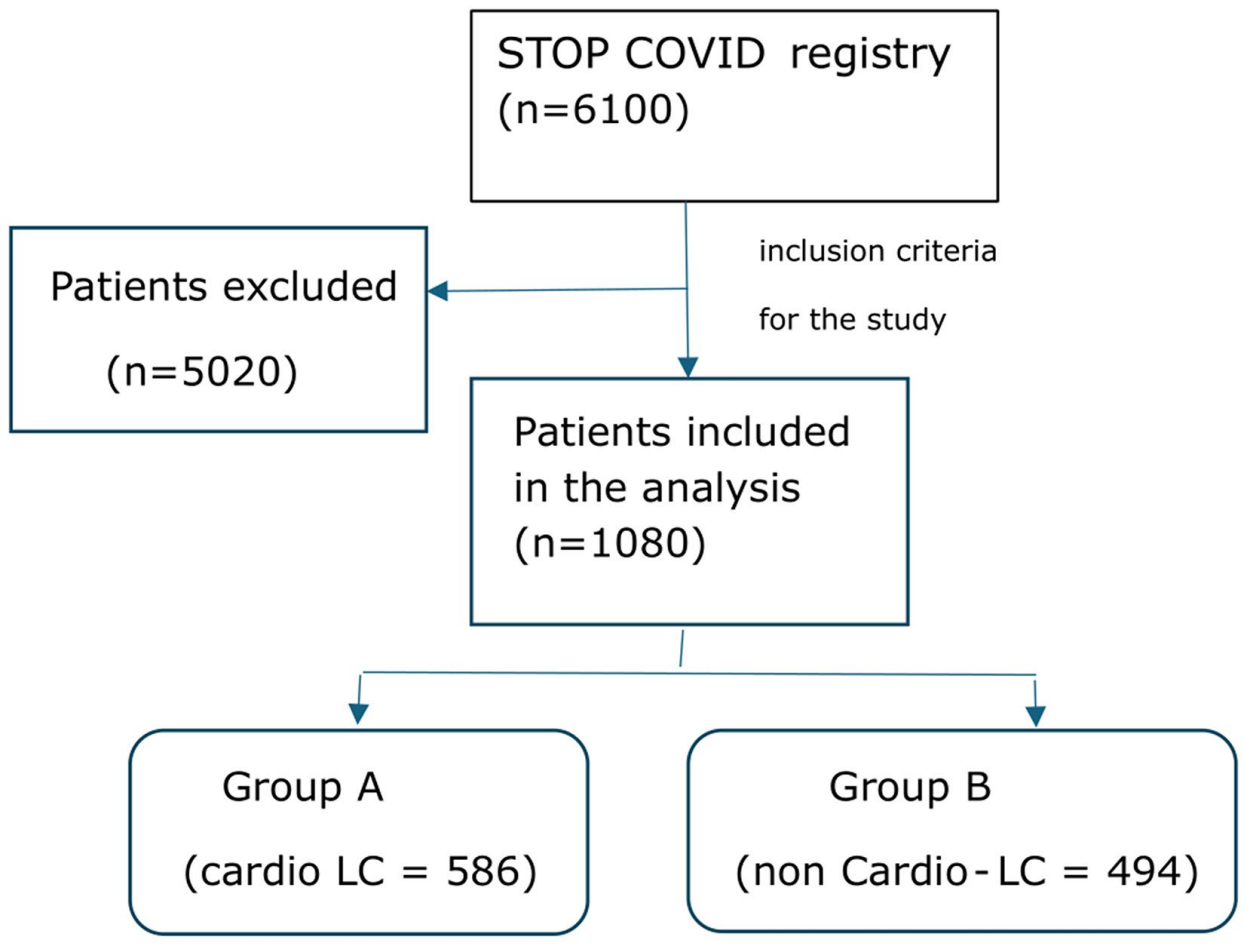
Study flowchart

Inclusion criteria for the above analysis included patients who have made a stationary visit to a doctor’s office within 3–6 months of contracting COVID-19 (from 6 weeks after the end of the acute phase of infection) confirmed by a PCR and/or antigen test, in accordance with the legal regulations in a given period). Inclusion criteria for the analysis included: age ≥ 18 years, confirmed COVID-19, informed consent to participate in the study, and lack of previously diagnosed cardiovascular diseases such as heart failure, condition after heart attack, condition after stroke, condition after cardiac surgery, and coronary heart disease. Failure to meet the above criteria excluded the patient from participating in further analysis (Fig. 1).

### Methodology

During the visit, patients were asked about sociodemographic variables and completed a health questionnaire that analyzed the occurrence of specific clinical symptoms. Chronic diseases were also assessed, including hypertension, diabetes, asthma, chronic obstructive pulmonary disease (COPD), hypothyroidism, and cardiovascular diseases (heart failure, coronary artery disease, condition after cardiosurgery and after a heart attack, etc.). Because severe hypertension (grade 3: ≥180/≥110 mmHg) may affect cardiac function, patients with hypertension were included in the study: grade 1 (mild), 140—159/90—99 mmHg; grade 2 (moderate), 160—179/100—109 mmHg. Additionally, patients were evaluated regarding anthropometric measurements (weight and height), on the basis of which the Body Mass Index (BMI) was calculated. The status of vaccination against COVID-19 was also assessed; a vaccinated patient was considered an individual who received the basic vaccination regimen before COVID-19. Full vaccination status included:

1 dose of Vaccine Jansen or.

2 doses of Vaxzevria.

2 doses of Comirnaty.

2 doses of Spikevax.

Booster doses were not analyzed. Moreover, from June 2022 patients had to complete the Patient Health Questionnaire-2 (PHQ-2) and Generalized Anxiety Disorder 2-item (GAD-2).

The PHQ-2 psychometric scale is a screening tool for the initial diagnosis of depression, which consists of two questions based on the original PHQ-9 scale while maintaining its psychometric properties. Patients can get from 0 to 6 points. The selected cut-off point was 3, per the recommendations of the authors of the tool. Above this value, the presence of depression should be suspected [37].

The GAD-2 scale is a shortened version of the GAD-7 scale used for screening the diagnosis of anxiety. The scale consists of two questions and a patient can get from 0 to 3 points. The selected cut-off point was 3, in accordance with the original version of the scale. Above this value, the presence of anxiety should be suspected [38].

As part of the visit, the patient underwent additional tests, which included ambulatory blood pressure monitoring (ABPM), Holter ECG and Echocardiography. Echocardiography was performed by a cardiologist. The examination was performed under the recommendations of the American Society of Echocardiography (ASE) and the European Society of Cardiovascular Imaging (EACVI) [39]. Standard projections necessary for a complete echocardiographic examination were recorded: parasternal and apical–four-chamber, two-chamber, and three-chamber, as well as a modified apical projection for the right ventricle. On their basis, the regional contractility in 17 segments of the left ventricular muscle and the contractile function of the right ventricle were visually analyzed. Based on the modified Simpson method, quantitative measurements of left ventricular function included end-systolic and end-diastolic volumes and left ventricular ejection fraction (EF). Quantitative assessment of the right ventricular systolic function was based on the evaluation of the tri-cuspid annular systolic excursion amplitude (TAPSE) and the measurement of the maximum myocardial systolic velocity S’ of the tricuspid annulus/basal segment of the RV free wall, established using tissue echocardiography (TDE). 24-hour Holter ECG monitoring was performed using PocketECG III (Medicalgorithmics Unified Arrhythmia Diagnostic System, Warsaw, Poland).

The diagnostic tools used in our study play a crucial role in ensuring the reliability of findings in the context of long COVID. The PHQ-2 and GAD-2 questionnaires, used for the rapid assessment of depression and anxiety, help identify mental health issues that are common among long COVID patients, thereby improving diagnostic accuracy and enabling quicker intervention. The ability of these questionnaires to swiftly diagnose mental health problems is important, as these symptoms can affect health outcomes and quality of life [40]. Blood pressure monitoring using ABPM allows for a more accurate assessment of hypertension, which is often a complication associated with SARS-CoV-2 infection [41, 42]. Meanwhile, Holter ECG is useful for detecting arrhythmias that may occur in patients after COVID-19 [43], and echocardiography is a vital tool in diagnosing myocardial damage, such as cardiomyopathy, enabling monitoring of heart function and preventing further complications [44]. All these tools allow for a comprehensive evaluation of patients’ health, contributing to more accurate results in long COVID research.

### Statistical analysis

The analyzed variables were qualitative and quantitative. The normality of distribution was assessed using the Shapiro-Wilk test. A comparison of quantitative variables was performed using the nonparametric Mann-Whitney U test. A comparison of qualitative variables was performed using the Chi-square test. In addition, to evaluate the relationship between ABPM, Holter ECG and echocardiography and PHQ-2 and GAD-2 scale scores, multivariate logistic regression analysis was performed, where the dependent variable was GAD-2 scale score ≥ 3 points, and independent variables included ABPM, Holter ECG and echocardiography scores. An analogous model included the dependent variable in the form of a PHQ-2 scale score ≥ 3 points. Statistica 13.0 (StatSoft, Tulsa, Oklahoma, United States) was used for calculations. In all tests, the level of statistical significance was *p* < 0.05.

## Results

### Characteristics of the group included in the study

The study included 1080 patients with a mean age of 56.9 ± 13.3 years. The vast majority were women (68.9%). Patients vaccinated against COVID-19 constituted 75.2% of the study group. The most common chronic diseases were hypertension (44.4%) and lipid disorders (18.0%). More than half of patients (53.1%) were affected during the dominance of the Omicron variant. A detailed summary of the study group is presented in Table 1.

**Table 1:** Characteristics of the study group, including division into patients with and without diagnosed cardio long COVID

Variable		The whole group *N*(%) M ± SD	Group A *N*(%) M ± SD	Group B *N*(%) M ± SD	*p*
Sex	Male	336 (31.1)	157 (46.7)	179 (53.3)	< 0.001
	Female	744 (68.9)	429 (57.7)	315 (42.3)	
Age		56.9 ± 13.3	56.9 ± 13.4	57.2 ± 13.1	0.595
BMI		27.8 ± 5.4	27.8 ± 5.6	27.8 ± 5.3	0.719
COVID-19 vaccination		812 (75.2)	457 (56.3)	355 (43.7)	0.020
Hypertension		480 (44.4)	253 (52.7)	227 (47.3)	0.360
DM		124 (11.5)	60 (48.4)	64 (51.6)	0.163
Hyperlipidemia		194 (18.0)	113 (58.3)	81 (41.7)	0.218
Asthma		104 (9.6)	66 (63.5)	38 (36.5)	0.047
COPD		17 (1.6)	12 (70.6)	5 (29.4)	0.173
Hypothyroidism		170 (15.7)	101 (59.4)	69 (40.6)	0.108
COVID-19 period	PreOmicron	507 (46.9)	274 (54.0)	233 (46.0)	0.893
	Omicron	573 (53.1)	312 (54.5)	261 (45.5)	

BMI, Body Mass Index; COPD, chronic obstructive pulmonary disease; DM, diabetes mellitus; N– number; M– Mean; SD– standard deviation, LC– long COVID

### Cardio long COVID

At least one of the analyzed symptoms occurred in 586 patients (54.3%), with the most common being fatigue (38.9%). Palpitations occurred in 17.6% of patients, whereas 19 patients declared episodes of syncope during the analyzed period. Most often, patients had 1 of these symptoms (45.7%). All investigated symptoms occurred in 6 patients (0.6%). A detailed summary of these results is presented in Table 2.

**Table 2:** Cardio long COVID symptoms in the analyzed group

Symptoms		*N* (%) M ± SD
Fatigue		420 (38.9)
Fainting		19 (1.8)
Dyspnoea		120 (11.1)
Chest pain		126 (11.7)
Heart palpitations		190 (17.6)
At least one of the cardiac symptoms analyzed		586 (54.3)
Number of symptoms	0	490 (45.7)
	1	399 (36.9)
	2	117 (10.8)
	3	44 (4.1)
	4	20 (1.9)
	5	6 (0.6)

N– number; M– Mean; SD– standard deviation

### Supplementary examination

The comparative analysis between patients with and without cardio-LC showed no differences in ABPM, Holter ECG, or echocardiograph. The detailed summary is presented in Table 3.

**Table 3:** Comparison of specific ABPM, Holter ECG and echocardiography parameters between patients with and without diagnosed cardio long COVID

Variables		The whole group *N*(%) M ± SD	Group A *N*(%) M ± SD	Group B *N*(%) M ± SD	*p*
ABPM	Systolic	124.7 ± 13.5	124.3 ± 13.3	125.2 ± 13.9	0.411
	Diastolic	73.7 ± 8.7	73.5 ± 8.2	73.8 ± 9.3	0.946
24-h Holter ECG	HR	75.3 ± 9.5	75.5 ± 9.6	75.1 ± 9.4	0.458
	VES	187.3 ± 1038.3	201.8 ± 1039.9	170.0 ± 1037.9	0.904
	Runs > 3	21 (1.9)	11 (55.0)	10 (45.0)	0.553
	NSVT	20 (1.8)	9 (45.0)	11 (55.0)	0.401
	SVES	119.4 ± 551.9	106.9 ± 488.3	134.3 ± 619.1	0.053
Echocardiography	Ao	31.1 ± 4.1	31.1 ± 3.9	31.0 ± 4.3	0.841
	EF	59.0 ± 4.1	59.1 ± 4.1	58.9 ± 4.0	0.561
	TAPSE	25.2 ± 2.4	25.1 ± 2.3	25.3 ± 2.5	0.321
	Any contraction abnormalities	81 (7.5)	50 (61.7)	31 (38.3)	0.161

Abbreviations: LC– long COVID; HE– heart rate; ECG, electrocardiogram; HR, heart rate; NSVT, nonsustained ventricular tachycardia; SVES, supraventricular extrasystole; VES, ventricular extrasystole; ABPM - Ambulatory Blood Pressure Monitoring; Ao– Aorta diameter; EF - Ejection Fraction; TAPSE - Tricuspid annular plane systolic excursion; Runs > 3 number or episodes with more than 3 consecutive ventricular extrasystoles

However, comparing patients with or without the feeling of heart palpitations showed that the mean value of ventricular extrasystole (VES) was higher in the former group (*p* = 0.011). Interestingly, these patients had lower average blood pressure values, both systolic (*p* = 0.001) and diastolic (*p* = 0.028). A detailed summary is presented in Table 4.

**Table 4:** Comparison of additional test results between patients with and without perceived heart palpitations

Variable		Heart palpitations *N*(%) M ± SD	Lack of heart palpitations *N*(%) M ± SD	*p*
ABPM	Systolic	121.7 ± 13.6	125.4 ± 13.5	**0.001**
	diastolic	72.3 ± 8.3	73.9 ± 8.8	**0.028**
24-h Holter ECG	HR	76.4 ± 9.9	75.1 ± 9.4	0.114
	VES	190.3 ± 896.3	186.6 ± 1066.8	**0.011**
	Runs > 3	1.63 ± 10.1	3.3 ± 71.5	0.149
	NSVT	4 (2.1)	186 (97.8)	0.778
	SVES	131.3 ± 461.1	116.9 ± 569.6	0.135
Echocardiography	Ao	30.6 ± 3.6	31.2 ± 4.2	**0.044**
	EF	59.5 ± 4.6	58.9 ± 3.9	**0.012**
	TAPSE	25.3 ± 2.3	25.2 ± 2.4	0.346
	Any contraction abnormalities	8 (4.2)	182 (95.8)	0.058

Abbreviations: HE– heart rate; ECG, electrocardiogram; HR, heart rate; NSVT, nonsustained ventricular tachycardia; SVES, supraventricular extrasystole; VES, ventricular extrasystole; ABPM - Ambulatory Blood Pressure Monitoring; Ao– Aorta diameter; EF - Ejection Fraction; TAPSE - Tricuspid annular plane systolic excursion; Runs > 3 number or episodes with more than 3 consecutive ventricular extrasystoles

### Mental condition

PHQ-2 and GAD-2 were assessed in 504 patients. Among this group, 290 (57.4%) declared the occurrence of at least 1 analyzed symptom in the period of 3–6 months after the end of SARS-CoV-2 infection. Comparing cardio Long COVID patients with and without cardiological symptoms, significant differences were found in the mean values of the PHQ-2 and GAD-2 scales, as well as in the percentage of responses indicating an increased risk of anxiety or depression. The detailed summary is presented in Table 5.

**Table 5:** Comparison of GAD-2 and PHQ-2 scale scores between patients with and without cardio long COVID symptoms

	The whole group (*N* = 504) N(%) M ± SD	Group A (*N* = 290) N(%) M ± SD	Group B (*N* = 214) N(%) M ± SD	*p*
PHQ-2 ≥ 3 points	156 (30.1)	103 (35.5)	53 (24.8)	**0.009**
PHQ-2 M ± SD	1.92 ± 1.8	2.06 ± 1.8	1.74 ± 1.7	**0.022**
GAD-2 ≥ 3 points	151 (29.9)	104 (35.9)	47 (22.0)	**0.008**
GAD-2 M ± SD	2.02 ± 1.7	2.27 ± 1.8	1.69 ± 1.6	**< 0.001**

**GAD-2 Generalized Anxiety Disorder 2-item; PHQ-2 Patient Health Questionnaire-2**, LC– long COVID, M– Mean; SD– standard deviation,

Multivariate logistic regression analysis evaluated the relationship between ABMP scores and PHQ-2 and GAD-2 scale scores. It was shown that subjects with PHQ-2 ≥ 3 points had higher heart rates. Detailed data are shown in Table 6.

**Table 6:** Results of multivariate logistic regression analysis evaluating the relationship between ABPM, Holter ECG and echocardiography parameters and PHQ-2 and GAD-2 scale scores

Variable		PHQ-2 ≥ 3 points		GAD-2 ≥ 3 points	
		OR [95Cl]	*p*	OR [95Cl]	*p*
ABPM	Systolic	0.98 [0.96, 1.01]	0.248	0.98 [0.96, 1.02]	0.239
	diastolic	0.99 [0.97, 1.03]	0.925	1.00 [0.97, 1.04]	0.979
24-h Holter ECG	HR	1.02 [1.01, 1.05]	**0.012**	1.01 [0,99, 1.03]	0.293
	VES	0.99 [0.99, 1.00]	0.371	0.99 [0.99, 1.01]	0.176
	Runs > 3	0.74 [0.14, 3/95]	0.733	3.81 [0.21, 6.43]	0.358
	NSVT	0.98 [0.96, 1.02]	0.754	0.31 [0.01, 8,71]	0.491
	SVES	0.99 [0.98, 1.01]	0.501	0.99 [0.98, 1.00]	0.778
Echocardiography	Ao	0.94 [0.88, 1.01]	0.063	0.95 [0.89, 1.00]	0.063
	EF	0.93 [0.86, 1.01]	0.106	**0.98 [0.91**,** 1.06]**	**0.633**
	TAPSE	1.04 [0.95, 1.13]	0.367	1.04 [0.96, 1.14]	0.321
	Any contraction abnormalities	0.55 [0.17, 1.81]	0.329	0.73 [0.22, 2.31]	0.587

Abbreviations: OR– odds ratio; HE– heart rate; ECG, electrocardiogram; HR, heart rate; NSVT, nonsustained ventricular tachycardia; SVES, supraventricular extrasystole; VES, ventricular extrasystole; ABPM - Ambulatory Blood Pressure Monitoring; Ao– Aorta diameter; EF - Ejection Fraction; TAPSE - Tricuspid annular plane systolic excursion; Runs > 3 number or episodes with more than 3 consecutive ventricular extrasystoles

## Discussion

This study aimed to investigate the clinical features of patients with persistent cardiological symptoms occurring within 3 to 6 months after COVID-19. The study found that people with palpitations had more excessive ventricular extrasystoles than patients without these symptoms. Additionally, it was observed that individuals with persistent cardiac symptoms after COVID-19 have a much worse mental condition. Long COVID is still not fully understood and although the spread of SARS-CoV-2 diminishes, patients experiencing persistent symptoms are being reported worldwide [8]. The pathophysiology of Long COVID remains elusive due to the diverse symptoms and affected organs, hampering the development of effective treatment strategies [45]. A notable rise in cardiovascular disease was noted among individuals who survived COVID-19, including those who did not require hospitalization [46]. Huseynov et al. indicate that on average 30% of patients who have recovered from COVID-19 experience persistent cardiopulmonary symptoms (including dyspnea, palpitations, reduced physical capacity, and cardiac arrhythmias) continuing for weeks or even months after the acute SARS-CoV-2 infection [47].

The present study indicates that Long COVID with cardiovascular complications is manifested more frequently among women, asthmatics, and vaccinated individuals. Cohen and colleagues showed that women are more likely to develop Long COVID and to have related activity limitations [48]. Similar observations were noted by Bai et al. [49] and Fernández-de-Las-Peñas et al. [50]. Women exhibit a slower decline in heart rate after physical exertion and experience a decrease in total lung capacity. Overall exercise tolerance in women may be caused by persistent cardiopulmonary abnormalities following a SARS-CoV-2 infection [51]. Cardiovascular diagnostics is suggested for Long COVID patients [52]. Asthma is linked with cardiovascular disease, and individuals affected by conditions often face an elevated risk of mortality [53]. Wolff et al. highlighted that pre-existing asthma, as observed in a hospital-based population, elevates the risk of Long COVID. Nonetheless, the authors noted that the conclusion was drawn from low-certainty evidence concerning population selection and the measurement of exposure or outcomes. Lee et al. acknowledged the increased likelihood of developing new-onset asthma following a COVID-19 infection [54]. Dolby et al. concluded that individuals with poorly controlled or severe asthma faced a higher risk of hospitalization due to COVID-19 compared to those without asthma. However, this association did not hold when examining mild or well-controlled asthma [55]. Previously, we reported that vaccination is ineffective in preventing Long COVID [56], which aligns with literature data [57]. Although mRNA vaccines can cause cardiovascular complications, Akhtar et al. indicated the vaccination is generally cardioprotective, with the rate of myopericarditis being lower than after SARS-CoV-2 infection [58]. Paknahad et al. concluded that the benefits of COVID-19 vaccination for personal and public health outweigh the modest cardiac risk, typically resolving within days or weeks [59]. The mechanisms regarding the relationship between vaccination and long COVID are not known. Recent data suggest that anti-idiotype antibodies mimic the virus, which arise both after infection and after vaccination, could potentially explain some of the long COVID-19 symptoms. Hence, hypotheses have emerged suggesting that vaccination through stimulation of the immune response may affect long COVID symptoms [60]. This could explain our data, where vaccinated patients were more likely to have symptoms after surviving COVID-19. However, as mentioned earlier, data evaluating the relationship between vaccination and long COVID are inconclusive. Part of the study shows that immunization reduces the risk of developing complications of the disease, part shows that it can exacerbate them, and part shows no relationship. The reasons for such discrepancies may be many, ranging from the lack of a uniform definition of long COVID, the considerable ethnic diversity of the analyzed groups, and finally the lack of assessment of many other factors that may affect the final results, such as the clinical condition of the patient, the severity of the infection, the use of chronic drugs and medications during the infection, or the type and number of doses of vaccination administered. Nevertheless, Lam et al. indicated that patients who completed their vaccination schedule or received a booster dose had a reduced risk of adverse health outcomes, including major cardiovascular diseases and all-cause mortality, compared to unvaccinated individuals or those with incomplete vaccination [61]. It is necessary to carry out further research with special attention to the mechanisms underlying the long COVID syndrome in order to unambiguously establish the relationship between vaccination and disease [62–64]. Investigating the influence of booster doses on Long COVID symptoms is advised, especially in larger cohorts. Studies should also assess the long-term impact of vaccination on cardiovascular symptoms and mental health to clarify the full spectrum of benefits and risks.

In particular, as mentioned earlier, the mechanisms underlying long COVID are not fully understood. Currently, theories of the formation of long COVID mainly focus on the immune response, the persistence of inflammation or even the survival of the virus in the body [45, 65]. However, when it comes to the mechanisms leading to the development of cardiovascular complications, one theory is that the receptors for angiotensin-converting enzyme 2 (ACE-2) may be crucial. Their high expression in COVID-19 patients may lead to hyperactivation of the renin-angiotensin-aldosterone axis, resulting in electrolyte disturbances and dysregulation of fluid homeostasis. This mechanism may underlie the development of hypertension and its subsequent sequelae, including arrhythmias and the development of heart failure [66, 67]. Metabolic syndrome and obesity may also promote cardiac changes in long COVID [68]. Future studies could focus on investigating the bidirectional relationship between mental health and cardiovascular symptoms in Long COVID patients. Potential mechanisms linking mental health and cardiovascular symptoms in Long COVID may include chronic inflammation, autonomic nervous system dysregulation, and endothelial dysfunction. Prolonged stress and anxiety can lead to elevated cortisol levels, which may exacerbate hypertension and arrhythmias. Additionally, shared pathways such as oxidative stress and immune dysregulation could further explain the interplay between these conditions, which is critical for developing holistic and integrated treatment strategies.

The lack of significant results in key cardiovascular parameters between patients with and without cardiac symptoms (cardio-LC vs. non-cardio-LC) noted in our study may be due to two factors: (1) some of these complications may be asymptomatic; (2) The complications concern damage to the vascular endothelium and/or myocytes of the cardiac muscle but in such a short time this may not yet lead to statistically significant differences in standard cardiovascular tests (ECHO, Holter ECG, Holter RR). Some publications show that after a longer period of observation (1–2 years), patients with LC develop, e.g., acute coronary syndromes [69]. Although no differences were noted in the analysis of symptoms common to cardio-LC and non-cardio-LC, the differences appear when we compare individual symptoms associated with individual tests (heart palpitations, ventricular arrhythmia). In addition, the symptoms that we classify as cardio-LC may be common to other long COVID complications, e.g., chronic fatigue syndrome or pulmonary complications [69]. This indicates that a patient with cardio-LC symptoms should be comprehensively diagnosed by multi-specialist clinical teams. The present study indicates that patients complaining about heart palpitations had higher ejection fraction and more ventricular extrasystoles but lower systolic and diastolic blood pressure. Magnetic resonance-cardiopulmonary exercise testing by Brown et al. revealed significantly higher resting LVEF (left ventricular ejection fraction) among post‐COVID patients relative to healthy controls [70]. Available data describe heart failure following myocarditis in Long COVID as impaired LVEF, diastolic dysfunction, and decreased right ventricle function [71]. Tamariz found that dysfunction of the left ventricle is common among Long COVID patients [72]. Puntmann et al. conducted cardiac assessments among COVID-19 patients with no previous cardiac disease. At follow-up (329 days after infection), 57% of participants had persistent cardiac symptoms. A significantly higher right ventricular ejection fraction was noted [73]. Chlabicz et al. acknowledged that the infection influenced the size of all heart chambers and the root of the aorta [74]. Podrug et al. outlined the predictors of the alterations in systemic hemodynamic parameters and arterial stiffness [75]. Stulova et al. examined cases of acute respiratory infections and/or other viral diseases complicated by myocarditis, indicating that 42.3% of patients exhibited recurrent and complicated ventricular extrasystoles on resting electrocardiograms, with 89% of these individuals having fibrous lesions in the pericardium [76]. Our previous research indicated that cardiac arrhythmias (AF– atrial fibrillation, SVES– supraventricular extrasystoles, VES– ventricular extrasystoles) observed on electrocardiograms were independent predictors of myocardial dysfunction following COVID-19 infection [34]. Cardiovascular disease as part of Long COVID was systematically reviewed by Tsampasian and colleagues [24]. Ramos et al. demonstrated that abnormal left ventricular global systolic longitudinal strain (LV-GLS), a measure of LV systolic function [77], is significantly associated with the occurrence of arrhythmias requiring intervention [78]. Moreover, Ingul et al. noted diastolic dysfunction occurring twice as frequently in patients three months post-hospitalization for COVID‐19 compared to matched controls; cardiac arrhythmias were prevalent after hospitalization, with premature ventricular beats detected in every fifth patient [79]. It has been confirmed that COVID-19 patients with cardiac arrhythmia have a higher frequency of comorbidities, including cardiovascular ones [80, 81]. Lastly, Wang et al. observed an elevated risk of atrial fibrillation, myocarditis, ischemic heart disease, and heart failure among COVID-19 survivors. These individuals had a higher 12-month risk of developing incidental cardiovascular diseases compared to non-COVID-19 controls [82]. These findings support the need for continued cardiovascular surveillance in post-COVID patients, particularly those with arrhythmias, to detect and manage any long-term complications.

Moreover, changes in the cardiovascular symptom profile in COVID-19 patients, associated with the dominance of a specific variant, have significant implications for clinical practice. Before the Omicron variant, where cardiovascular symptoms were more frequent and varied (such as chest pain, shortness of breath, or arrhythmias), patients required intensive monitoring for cardiovascular complications such as heart attacks, heart failure, or stroke risk [34]. In this context, physicians had to implement detailed diagnostics and more frequent hospitalizations, especially for those with comorbidities such as hypertension, diabetes, or coronary artery disease [83]. In contrast, patients infected with Omicron exhibited a milder course of the disease with fewer cardiovascular symptoms [84], which can be explained by a lower inflammatory response and Omicron’s greater tropism for the upper respiratory tract. Nevertheless, patients with or without comorbidities should still undergo regular cardiological checks to detect potential later complications, such as long-term cardiovascular symptoms [46, 83]. Clinical practice should also include broad patient education, informing them about potential cardiovascular symptoms after COVID-19 infection, regardless of the dominant variant, and emphasizing the importance of early reporting of any concerning symptoms, which allows for quicker response and appropriate therapy adjustments [46].

Comparing cardio Long COVID patients with and without cardiological symptoms, significant differences were found in the mean values of the PHQ-2 and GAD-2 scales, as well as in the percentage of responses indicating an increased risk of anxiety or depression. The present study indicates that Long COVID patients with cardiovascular symptoms are more commonly depressed and anxious. This complements the Atchison et al. study, where disparities in PHQ-2 scores were observed among patients with ongoing persistent COVID-19 compared to those with resolved short/persistent COVID-19 or asymptomatic disease course. Adults with positive PHQ-2 were notably more prone to experiencing persistent symptoms [85]. As assessed by PHQ-2 and GAD-2, Loftis and colleagues confirmed that COVID-19 severity was associated with increased depression and, to a lesser extent, anxiety [86]. Kim et al. certified that patients with Long COVID had higher PHQ-9 and GAD-7 scores than those without persistent symptoms [87]. Matsumoto and others noted higher PHQ-9 and GAD-7 scores among patients with post-COVID relative to non-infected participants and individuals without post-COVID [88]. Klein et al. indicated that GAD-7 and PHQ-2 were elevated in individuals experiencing persistent symptoms following acute infection compared to healthy controls and previously infected patients without persistent symptoms [89]. Morrow et al. found that 28–60 days post-discharge, COVID-19 patients exhibited higher PHQ-4 scores and displayed evidence of cardio-renal involvement and activation of the hemostasis pathway [90]. Jimeno-Almazán et al. evaluated the impact of an 8-week supervised exercise regimen compared to no intervention in individuals with post-COVID-19 condition, revealing that patients engaging in exercise showed improved depression symptoms [91]. The negative impact of Long COVID on mental condition indices, such as anxiety and depression, is consistent with a broader understanding of the quality-of-life implications experienced by those affected [92]. Anxiety was confirmed as a risk factor for cardiovascular disease [93], so it is no wonder that the clinical guidelines on cardiovascular disease prevention by the European Society of Cardiology also refer to psychiatric and psychosocial factors [94]. In research by Peng et al., it was found that 60% of COVID-19 survivors experienced mental distress even a year after discharge, with uncontrollable and excessive worry being among the most prominent symptoms [95]. Mental health difficulties have been reported in COVID-19 survivors, which may involve physiological and psychological factors [96].

Long-term stress, depression, and anxiety in the course of long COVID can significantly affect the cardiovascular health of patients [97, 98]. Disorders of the autonomic nervous system, caused by both the direct impact of the virus and chronic stress associated with the disease, can lead to dysfunctions in the regulation of cardiovascular functions, manifested by tachycardia, orthostatic hypotension, or other cardiac problems [99, 100]. Understanding these mechanisms is crucial to effectively support patients, taking into account both physical and mental aspects of their health.

Future studies should consider the clinical implications of these cardiovascular findings, particularly in terms of early detection of Long COVID. Comparing clinical outcomes between hospitalized and non-hospitalized patients could offer valuable insights for developing targeted interventions. Exploring the role of genetic predispositions in the development of Long COVID-related cardiovascular and mental health symptoms is needed. Additionally, studies could investigate the efficacy of non-pharmacological interventions, such as mindfulness-based therapies or exercise programs, in mitigating cardiovascular symptoms and ensuring mental health. The impact of social determinants such as socioeconomic status and access to care warrants deeper investigation. Finally, longitudinal studies are needed to assess the long-term trajectories of mental and cardiovascular health in Long COVID patients.

### Limitations of the study

The authors are aware of the limitations of the present study, which is undoubtedly the lack of assessing markers such as troponin to evaluate the degree of myocardial damage. The lack of long-term follow-up and retrospective nature are other drawbacks, with the latter entailing a risk of memory error that affects the reliability of estimated symptoms’ frequency. The recruitment of patients to the study may have encountered some errors and limitations that could have affected the reliability of the results. There may have been a selection of the samples, e.g., by limiting participants to a certain age group or excluding people with other comorbidities (other than those analyzed in the study). In addition, not all patients had the GAD-2 and PHQ-2 psychometric questionnaires performed. It should also be noted that these questionnaires are for screening purposes, so a clinical diagnosis is further required. In addition, some variables in multivariate regression analysis lack statistical significance (Table 6). Similarly, the *p*-values for some comorbidities (Table 1) are non-significant, which could impact the strength of conclusions. ECHO parameters (MASPE, STRAIN) were not evaluated. We did not analyze other chronic conditions that could have affected the final results. Moreover, pulmonary pathologies may have caused some symptoms such as fatigue or shortness of breath. Medications used during the acute phase of COVID-19, such as remdesivir, paxlovid, or corticosteroids, were not evaluated but could affect cardiac symptoms. Our research did not include data on booster doses against COVID-19, which may influence the interpretation of symptom persistence and cardiovascular outcomes, especially since we noticed more frequent cardiac symptoms among vaccinated individuals while vaccination is overall cardioprotective. The analyzed group is not representative of those who have undergone COVID-19 since patients volunteered for the above registry. Ultimately, there was a lack of knowledge about cardiac problems before COVID-19. In addition, the study is also limited by the lack of more data on echocardiography and laboratory tests such as troponin. Another limitation is the lack of data on hospitalizations between COVID-19 and the follow-up visit. However, to reduce the likelihood, only patients without prior cardiac diagnoses were included in the final analysis.

## Conclusion

People with palpitations had more excessive ventricular extrasystoles than patients without these symptoms. Moreover, individuals with persistent cardiac symptoms after COVID-19 have a much worse mental condition. Patients with long COVID require a comprehensive approach that takes into account both cardiac problems and the mental health of patients. Physicians should regularly monitor cardiac symptoms, such as palpitations and excessive ventricular contractions, by ordering additional diagnostic tests (ECG, Holter). In addition, due to the high frequency of mental disorders among patients with long COVID, it is also necessary to assess the mental state, including depression and anxiety. A holistic approach to treatment, combining cardiology, psychological and rehabilitation care, is crucial to improving the quality of life of patients. Long COVID is still not fully understood and further prospective studies with longer follow-up are necessary to understand the pathomechanisms and therapeutic options.

## Data Availability

The data presented in this study are available on request from the corresponding author.

## References

[1] World Health Organization. COVID-19 epidemiological update– 12 April 2024. Available online: https://www.who.int/publications/m/item/covid-19-epidemiological-update-edition-166 (accessed on 15 May 2024).

[2] Raport zakażeń koronawirusem (SARS-CoV-2). Available online: https://www.gov.pl/web/koronawirus/wykaz-zarazen-koronawirusem-sars-cov-2 (accessed on 15 May 2024).

[3] Cheng K, Wu C, Gu S, Lu Y, Wu H, Li C. WHO declares the end of the COVID-19 global health emergency: lessons and recommendations from the perspective of ChatGPT/GPT-4. Int J Surg. 2023;109(9):2859–62. 37246993 10.1097/JS9.0000000000000521 PMC10498859

[4] Makhluf H, Madany H, Kim K, Long COVID. Long-Term impact of SARS-CoV2. Diagnostics (Basel). 2024, 14(7). 10.3390/diagnostics14070711 PMC11011397 38611624

[5] Mahase E. Long Covid could be four different syndromes, review suggests. BMJ. 2020. 10.1136/bmj.m3981 33055076

[6] Golchin Vafa R, Heydarzadeh R, Rahmani M et al. The long-term effects of the Covid-19 infection on cardiac symptoms. BMC Cardiovasc Disord. 2023, 23(1). 10.1186/s12872-023-03322-8 PMC10243271 37280530

[7] O’Mahoney LL, Routen A, Gillies C et al. The prevalence and long-term health effects of long Covid among hospitalised and non-hospitalised populations: a systematic review and meta-analysis. eClinicalMedicine. 2023, 55. 10.1016/j.eclinm.2022.101762 PMC9714474 36474804

[8] Davis HE, McCorkell L, Vogel JM, Topol EJ. Long COVID: major findings, mechanisms and recommendations. Nat Rev Microbiol. 2023;21(3):133–46. 36639608 10.1038/s41579-022-00846-2 PMC9839201

[9] Bende F, Tudoran C, Sporea I et al. A multidisciplinary approach to evaluate the presence of hepatic and cardiac abnormalities in patients with Post-Acute COVID-19 Syndrome-A pilot study. J Clin Med. 2021, 10(11). 10.3390/jcm10112507 PMC8201250 34204032

[10] Higgins V, Sohaei D, Diamandis EP, Prassas I. COVID-19: from an acute to chronic disease? Potential long-term health consequences. Crit Rev Clin Lab Sci. 2021;58(5):297–310. 33347790 10.1080/10408363.2020.1860895

[11] Natarajan A, Shetty A, Delanerolle G et al. A systematic review and meta-analysis of long COVID symptoms. Syst Reviews. 2023, 12(1). 10.1186/s13643-023-02250-0 PMC10220332 37245047

[12] Tziolos N-R, Ioannou P, Baliou S, Kofteridis DP. Long COVID-19 Pathophysiology: What Do We Know So Far? Microorganisms. 2023, 11(10). 10.3390/microorganisms11102458 PMC10609046 37894116

[13] Babicki M, Kołat D, Kapusta J, et al. Prevalence and assessment of risk factors among Polish adults with post-COVID syndrome: 12-month follow-up study. Polish Archives of Internal Medicine; 2023. 10.20452/pamw.16512 37338234

[14] Lorente-Ros M, Das S, Elias J, Frishman WH, Aronow WS. Cardiovascular manifestations of the long COVID syndrome. Cardiol Rev. 2023; Publish Ahead of Print. 10.1097/CRD.0000000000000552 37071080

[15] Pierre V, Draica F, Di Fusco M, et al. The impact of vaccination and outpatient treatment on the economic burden of Covid-19 in the united States Omicron era: a systematic literature review. J Med Econ. 2023;26(1):1519–31. 37964554 10.1080/13696998.2023.2281882

[16] Tulimilli SV, Dallavalasa S, Basavaraju CG et al. Variants of severe acute respiratory syndrome coronavirus 2 (SARS-CoV-2) and vaccine effectiveness. Vaccines. 2022, 10(10). 10.3390/vaccines10101751 PMC9607623 36298616

[17] Dobrowolska K, Brzdęk M, Zarębska-Michaluk D, et al. Differences between the course of SARS-CoV-2 infections in the periods of the delta and Omicron variants dominance in Poland. Polish Archives of Internal Medicine; 2023. 10.20452/pamw.16403 36602857

[18] Nagy Á, Pongor S, Győrffy B. Different mutations in SARS-CoV-2 associate with severe and mild outcome. Int J Antimicrob Agents. 2021, 57(2). 10.1016/j.ijantimicag.2020.106272 PMC7755579 33347989

[19] Rzymski P, Pokorska-Śpiewak M, Jackowska T et al. Key considerations during the transition from the acute phase of the COVID-19 pandemic: A narrative review. Vaccines. 2023, 11(9). 10.3390/vaccines11091502 PMC10537111 37766178

[20] Tian D, Sun Y, Xu H, Ye Q. The emergence and epidemic characteristics of the highly mutated SARS-CoV‐2 Omicron variant. J Med Virol. 2022;94(6):2376–83. 35118687 10.1002/jmv.27643 PMC9015498

[21] Ntchana A, Shrestha S, Pippin M. Cardiovascular Complications of COVID-19: A Scoping Review of Evidence. Cureus. 2023. 10.7759/cureus.48275 PMC10695704 38054135

[22] Babicki M, Kołat D, Kałuzińska-Kołat Ż et al. The course of COVID-19 and long COVID: identifying risk factors among patients suffering from the disease before and during the Omicron-Dominant period. Pathogens. 2024, 13(3). 10.3390/pathogens13030267 PMC10975347 38535610

[23] Hedberg P, Nauclér P. Post–COVID-19 condition after SARS-CoV-2 infections during the Omicron surge vs the delta, alpha, and wild type periods in Stockholm, Sweden. J Infect Dis. 2024;229(1):133–36. 37665981 10.1093/infdis/jiad382 PMC10786247

[24] Tsampasian V, Bäck M, Bernardi M et al. Cardiovascular disease as part of long COVID: a systematic review. Eur J Prev Cardiol. 2024. 10.1093/eurjpc/zwae070 38381595

[25] Schulte E. 68. Untangling genetic risk factors of long Covid: work of the international Covid-19 host genetics initiative. Eur Neuropsychopharmacol. 2022, 63.

[26] Wang S, Quan L, Chavarro JE et al. Associations of depression, anxiety, worry, perceived stress, and loneliness prior to infection with risk of Post–COVID-19 conditions. JAMA Psychiatry 2022, 79(11). 10.1001/jamapsychiatry.2022.2640 PMC9453634 36069885

[27] Islam MS, Phu DH, Maneerattanasak S et al. Prevalence and factors associated with long COVID and mental health status among recovered COVID-19 patients in Southern Thailand. PLoS ONE. 2023, 18(7). 10.1371/journal.pone.0289382 PMC10389739 37523396

[28] Seighali N, Abdollahi A, Shafiee A et al. The global prevalence of depression, anxiety, and sleep disorder among patients coping with post COVID-19 syndrome (long COVID): a systematic review and meta-analysis. BMC Psychiatry. 2024, 24(1). 10.1186/s12888-023-05481-6 PMC10848453 38321404

[29] Engelmann P, Reinke M, Stein C, et al. Psychological factors associated with long COVID: a systematic review and meta-analysis. EClinicalMedicine. 2024;74:102756. 39764180 10.1016/j.eclinm.2024.102756 PMC11701445

[30] McDonagh TA, Metra M, Adamo M, et al. 2021 ESC guidelines for the diagnosis and treatment of acute and chronic heart failure. Eur Heart J. 2021;42(36):3599–726. 34447992 10.1093/eurheartj/ehab368

[31] Lim GB. Myocardial injury in patients with COVID-19. Nat Rev Cardiol. 2020;17(8):454. 32572194 10.1038/s41569-020-0408-6 PMC7306657

[32] Lee CCE, Ali K, Connell D et al. COVID-19-Associated cardiovascular complications. Diseases. 2021, 9(3). 10.3390/diseases9030047 PMC8293160 34209705

[33] Tudoran C, Tudoran M, Lazureanu VE et al. Factors influencing the evolution of pulmonary hypertension in previously healthy subjects recovering from a SARS-CoV-2 infection. J Clin Med. 2021, 10(22). 10.3390/jcm10225272 PMC8625017 34830554

[34] Kapusta J, Babicki M, Pieniawska-Smiech K, et al. Clinical and electrocardiographic correlates of myocardial dysfunction after COVID-19 in nonhospitalised patients in long-term follow-up. Data from the Polish long-covid cardiovascular study. J Med Virol. 2023;95(12):e29331. 38112151 10.1002/jmv.29331

[35] Szarpak L, Pruc M, Filipiak KJ, et al. Myocarditis: A complication of COVID-19 and long-COVID-19 syndrome as a serious threat in modern cardiology. Cardiol J. 2022;29(1):178–79. 34811716 10.5603/CJ.a2021.0155 PMC8890406

[36] Glowniak A, Wojewoda K, Tarkowski A. COVID-19 and long-COVID-19 syndrome related myocarditis: the heart rhythm matters. Cardiol J. 2023;30(1):165–66. 36790044 10.5603/CJ.a2023.0005 PMC9987533

[37] Kroenke K, Spitzer RL, Williams JBW. The patient health Questionnaire-2. Med Care. 2003;41(11):1284–92. 14583691 10.1097/01.MLR.0000093487.78664.3C

[38] Kroenke K, Spitzer RL, Williams JBW, Monahan PO, Löwe B. Anxiety disorders in primary care: prevalence, impairment, comorbidity, and detection. Ann Intern Med. 2007, 146(5). 10.7326/0003-4819-146-5-200703060-00004 17339617

[39] Lang RM, Badano LP, Mor-Avi V, et al. Recommendations for cardiac chamber quantification by echocardiography in adults: an update from the American society of echocardiography and the European association of cardiovascular imaging. J Am Soc Echocardiogr. 2015;28(1):1–e3914. 25559473 10.1016/j.echo.2014.10.003

[40] Sung S, Kim SH, Lee C, Kim Y, Bae YS, Chie EK. The association of acute signs and symptoms of COVID-19 and exacerbation of depression and anxiety in patients with clinically mild COVID-19: retrospective observational study. JMIR Public Health Surveill. 2023;9:e43003. 36645439 10.2196/43003 PMC9926346

[41] Wojciechowska W, Rajzer M, Kreutz R, et al. The impact of the COVID-19 pandemic on blood pressure control in patients with treated hypertension-results of the European society of hypertension study (ESH ABPM COVID-19 study). J Hypertens. 2024;42(12):2065–74. 39248094 10.1097/HJH.0000000000003752

[42] Wasim D, Alme B, Jordal S, et al. Characteristics of 24-hour ambulatory blood pressure monitoring in a COVID-19 survivor. Future Cardiol. 2021;17(8):1321–26. 33876965 10.2217/fca-2020-0235 PMC8056747

[43] Ceren I, Habip FB. How did coronavirus disease 2019 affect autonomic balance in young individuals? Analysis by heart rate variability. Rev Assoc Med Bras (1992). 2024;70(10):e20231789. 10.1590/1806-9282.20231789 PMC11444206 39356955

[44] Barosi A, Bergamaschi L, Cusmano I, Gasperetti A, Schiavone M, Gherbesi E. Echocardiography in COVID-19 pandemic: clinical findings and the importance of emerging technology. Card Electrophysiol Clin. 2022;14(1):71–8. 35221087 10.1016/j.ccep.2021.10.007 PMC8556576

[45] Castanares-Zapatero D, Chalon P, Kohn L, et al. Pathophysiology and mechanism of long COVID: a comprehensive review. Ann Med. 2022;54(1):1473–87. 35594336 10.1080/07853890.2022.2076901 PMC9132392

[46] Xie Y, Xu E, Bowe B, Al-Aly Z. Long-term cardiovascular outcomes of COVID-19. Nat Med. 2022;28(3):583–90. 35132265 10.1038/s41591-022-01689-3 PMC8938267

[47] Huseynov A, Akin I, Duerschmied D, Scharf RE. Cardiac arrhythmias in Post-COVID syndrome: prevalence, pathology, diagnosis, and treatment. Viruses. 2023, 15(2). 10.3390/v15020389 PMC9959721 36851603

[48] Cohen J, van der Meulen Rodgers Y. An intersectional analysis of long COVID prevalence. Int J Equity Health. 2023, 22(1). 10.1186/s12939-023-02072-5 PMC10717295 38093291

[49] Bai F, Tomasoni D, Falcinella C et al. Female gender is associated with long COVID syndrome: a prospective cohort study. Clinical Microbiology and Infection. 2022;28(4):611.e9-11.e16. 10.1016/j.cmi.2021.11.002 PMC8575536 34763058

[50] Fernández-de-las-Peñas C, Martín-Guerrero JD, Pellicer-Valero ÓJ et al. Female sex is a risk factor associated with Long-Term Post-COVID Related-Symptoms but not with COVID-19 symptoms: the LONG-COVID-EXP-CM multicenter study. J Clin Med. 2022, 11(2). 10.3390/jcm11020413 PMC8778106 35054108

[51] Baranauskas MN, Carter SJ. Evidence for impaired chronotropic responses to and recovery from 6-minute walk test in women with post‐acute COVID‐19 syndrome. Exp Physiol. 2021;107(7):722–32. 34761446 10.1113/EP089965 PMC8667649

[52] Gyöngyösi M, Alcaide P, Asselbergs FW, et al. Long COVID and the cardiovascular system—elucidating causes and cellular mechanisms in order to develop targeted diagnostic and therapeutic strategies: a joint scientific statement of the ESC working groups on cellular biology of the heart and myocardial and pericardial diseases. Cardiovascular Res. 2023;119(2):336–56. 10.1093/cvr/cvac115 PMC9384470 35875883

[53] Cazzola M, Hanania NA, Rogliani P, Matera MG. Cardiovascular disease in asthma patients: from mechanisms to therapeutic implications. Kardiologia Polska. 2023;81(3):232–41. 36739655 10.33963/KP.a2023.0038

[54] Lee H, Kim B-G, Chung SJ, et al. New-onset asthma following COVID-19 in adults. J Allergy Clin Immunology: Pract. 2023;11(7):2228–31. 37084939 10.1016/j.jaip.2023.03.050 PMC10116152

[55] Dolby T, Nafilyan V, Morgan A, Kallis C, Sheikh A, Quint JK. Relationship between asthma and severe COVID-19: a National cohort study. Thorax. 2023;78(2):120–27. 35354646 10.1136/thoraxjnl-2021-218629 PMC8983409

[56] Chudzik M, Babicki M, Kapusta J et al. Long-COVID clinical features and risk factors: A retrospective analysis of patients from the STOP-COVID registry of the PoLoCOV study. Viruses. 2022, 14(8). 10.3390/v14081755 PMC9415629 36016376

[57] Al-Aly Z, Bowe B, Xie Y. Long COVID after breakthrough SARS-CoV-2 infection. Nat Med. 2022;28(7):1461–67. 35614233 10.1038/s41591-022-01840-0 PMC9307472

[58] Akhtar Z, Trent M, Moa A, Tan TC, Fröbert O, MacIntyre CR. The impact of COVID-19 and COVID vaccination on cardiovascular outcomes. Eur Heart J Supplements. 2023;25(Supplement_A):A42–9. 10.1093/eurheartjsupp/suac123 PMC10021497 36937372

[59] Paknahad MH, Yancheshmeh FB, Soleimani A. Cardiovascular complications of COVID-19 vaccines: A review of case-report and case-series studies. Heart Lung. 2023;59:173–80. 36842342 10.1016/j.hrtlng.2023.02.003 PMC9905103

[60] Murphy WJ, Hamel MB, Longo DL. A possible role for Anti-idiotype antibodies in SARS-CoV-2 infection and vaccination. N Engl J Med. 2022;386(4):394–96. 34818473 10.1056/NEJMcibr2113694

[61] Lam ICH, Zhang R, Man KKC, et al. Persistence in risk and effect of COVID-19 vaccination on long-term health consequences after SARS-CoV-2 infection. Nat Commun. 2024;15(1):1716. 38403654 10.1038/s41467-024-45953-1 PMC10894867

[62] Thanachartwet V, Arjun MC, Singh AK et al. Characteristics and predictors of long COVID among diagnosed cases of COVID-19. PLoS ONE. 2022, 17(12). 10.1371/journal.pone.0278825 PMC9767341 36538532

[63] Fernández-de-las-Peñas C, Ortega-Santiago R, Fuensalida-Novo S, Martín-Guerrero JD, Pellicer-Valero OJ, Torres-Macho J. Differences in Long-COVID symptoms between vaccinated and Non-Vaccinated (BNT162b2 Vaccine) hospitalized COVID-19 survivors infected with the delta variant. Vaccines. 2022, 10(9). 10.3390/vaccines10091481 PMC9504977 36146560

[64] Wisnivesky JP, Govindarajulu U, Bagiella E, et al. Association of vaccination with the persistence of Post-COVID symptoms. J Gen Intern Med. 2022;37(7):1748–53. 35266128 10.1007/s11606-022-07465-w PMC8906626

[65] Mehandru S, Merad M. Pathological sequelae of long-haul COVID. Nat Immunol. 2022;23(2):194–202. 35105985 10.1038/s41590-021-01104-y PMC9127978

[66] Liu H, Wang Z, Sun H et al. Thrombosis and coagulopathy in COVID-19: current Understanding and implications for antithrombotic treatment in patients treated with percutaneous coronary intervention. Front Cardiovasc Med. 2021, 7. 10.3389/fcvm.2020.599334 PMC7847976 33537347

[67] Faghy M, Tuttiett E, Ryan D et al. COVID-19 and the long-term cardio-respiratory and metabolic health complications. Rev Cardiovasc Med. 2022, 23(2). 10.31083/j.rcm2302053 35229544

[68] Tudoran C, Tudoran M, Cut TG et al. The impact of metabolic syndrome and obesity on the evolution of diastolic dysfunction in apparently healthy patients suffering from Post-COVID-19 syndrome. Biomedicines. 2022, 10(7). 10.3390/biomedicines10071519 PMC9312435 35884823

[69] Fedorowski A, Fanciulli A, Raj SR, Sheldon R, Shibao CA, Sutton R. Cardiovascular autonomic dysfunction in post-COVID-19 syndrome: a major health-care burden. Nat Rev Cardiol. 2024;21(6):379–95. 38163814 10.1038/s41569-023-00962-3

[70] Brown JT, Saigal A, Karia N et al. Ongoing exercise intolerance following COVID-19: A magnetic Resonance–Augmented cardiopulmonary exercise test study. J Am Heart Association. 2022, 11(9). 10.1161/JAHA.121.024207 PMC9238618 35470679

[71] Rohun J, Dorniak K, Faran A, Kochańska A, Zacharek D, Daniłowicz-Szymanowicz L. Long COVID-19 myocarditis and various heart failure presentations: A case series. J Cardiovasc Dev Disease. 2022, 9(12). 10.3390/jcdd9120427 PMC9785067 36547424

[72] Tamariz L, Ryan M, Marzouka GR, Bast E, Klimas N, Palacio A. Cardiovascular risk factors predict who should have echocardiographic evaluation in long COVID. Echocardiography. 2024, 41(2).

[73] Puntmann VO, Martin S, Shchendrygina A, et al. Long-term cardiac pathology in individuals with mild initial COVID-19 illness. Nat Med. 2022;28(10):2117–23. 36064600 10.1038/s41591-022-02000-0 PMC9556300

[74] Chlabicz M, Dubatowka M, Jamiolkowski J et al. Enlarged dimensions of cardiac chambers and the root of the aorta as a significant cardiovascular impact of pandemic COVID-19: a population-based study. Eur J Prev Cardiol. 2023, 30(Supplement_1).

[75] Podrug M, Koren P, Dražić Maras E et al. Long-Term adverse effects of mild COVID-19 disease on arterial stiffness, and systemic and central hemodynamics: A Pre-Post study. J Clin Med. 2023, 12(6). 10.3390/jcm12062123 PMC10055477 36983124

[76] Stulova MA, Konstantinova EV. [Ventricular extrasystole as manifestation of viral myocarditis and myopericarditis in young patients]. Ter Arkh. 2007;79(12):28–34. 18220027

[77] Liu Y-W, Tseng C-C, Su C-T, et al. The prognostic value of left ventricular global peak systolic longitudinal strain in chronic peritoneal Dialysis patients. IJC Heart Vasculature. 2014;5:1–8. 28785605 10.1016/j.ijcha.2014.10.016 PMC5497151

[78] Ramos SZ, Putra HM, Locnen SA, Escasura G, Atutubo BK. Association of subclinical left ventricular systolic dysfunction detected on 2d echocardiogram global longitudinal strain and in-hospital adverse clinical outcomes in Covid 19 patients. Eur Heart J. 2023, 44(Supplement_2).

[79] Ingul CB, Grimsmo J, Mecinaj A et al. Cardiac dysfunction and arrhythmias 3 months after hospitalization for COVID-19. J Am Heart Association. 2022, 11(3). 10.1161/JAHA.121.023473 PMC9238505 35048715

[80] Peltzer B, Manocha KK, Ying X et al. Arrhythmic Complications of Patients Hospitalized With COVID-19. Circulation: Arrhythmia and Electrophysiology. 2020, 13(10). 10.1161/CIRCEP.120.009121 PMC7566289 32931709

[81] Coromilas EJ, Kochav S, Goldenthal I et al. Worldwide survey of COVID-19–Associated arrhythmias. Circulation: Arrhythmia Electrophysiol. 2021, 14(3). 10.1161/CIRCEP.120.009458 PMC7982128 33554620

[82] Wang W, Wang C-Y, Wang S-I, Wei JC-C. Long-term cardiovascular outcomes in COVID-19 survivors among non-vaccinated population: A retrospective cohort study from the TriNetX US collaborative networks. eClinicalMedicine. 2022, 53. 10.1016/j.eclinm.2022.101619 PMC9366236 35971425

[83] Guo T, Fan Y, Chen M, et al. Cardiovascular implications of fatal outcomes of patients with coronavirus disease 2019 (COVID-19). JAMA Cardiol. 2020;5(7):811–18. 32219356 10.1001/jamacardio.2020.1017 PMC7101506

[84] Babicki M, Kolat D, Kaluzinska-Kolat Z et al. The course of COVID-19 and long COVID: identifying risk factors among patients suffering from the disease before and during the Omicron-Dominant period. Pathogens. 2024, 13(3). 10.3390/pathogens13030267 PMC10975347 38535610

[85] Naidu SB, Shah AJ, Saigal A et al. The high mental health burden of long COVID and its association with on-going physical and respiratory symptoms in all adults discharged from hospital. Eur Respir J. 2021, 57(6). 10.1183/13993003.04364-2020 PMC8015645 33795319

[86] Loftis JM, Firsick E, Shirley K et al. Inflammatory and mental health sequelae of COVID-19. Compr Psychoneuroendocrinology. 2023, 15. 10.1016/j.cpnec.2023.100186 PMC10191701 37223650

[87] Kim Y, Bae S, Chang H-H, Kim S-W. Characteristics of long COVID and the impact of COVID-19 vaccination on long COVID 2 years following COVID-19 infection: prospective cohort study. Sci Rep. 2024, 14(1). 10.1038/s41598-023-50024-4 PMC10774352 38191556

[88] Matsumoto K, Hamatani S, Shimizu E, Käll A, Andersson G. Impact of post-COVID conditions on mental health: a cross-sectional study in Japan and Sweden. BMC Psychiatry. 2022, 22(1). 10.1186/s12888-022-03874-7 PMC8977559 35379224

[89] Klein J, Wood J, Jaycox JR, et al. Distinguishing features of long COVID identified through immune profiling. Nature. 2023;623(7985):139–48. 37748514 10.1038/s41586-023-06651-y PMC10620090

[90] Morrow AJ, Sykes R, McIntosh A, et al. A multisystem, cardio-renal investigation of post-COVID-19 illness. Nat Med. 2022;28(6):1303–13. 35606551 10.1038/s41591-022-01837-9 PMC9205780

[91] Jimeno-Almazán A, Franco‐López F, Buendía‐Romero Á, et al. Rehabilitation for post‐COVID‐19 condition through a supervised exercise intervention: A randomized controlled trial. Scand J Med Sci Sports. 2022;32(12):1791–801. 36111386 10.1111/sms.14240 PMC9538729

[92] Alacevich C, Thalmann I, Nicodemo C, de Lusignan S, Petrou S. Depression and anxiety during and after episodes of COVID-19 in the community. Sci Rep. 2023, 13(1). 10.1038/s41598-023-33642-w PMC10201488 37217539

[93] Allgulander C. Anxiety as a risk factor in cardiovascular disease. Curr Opin Psychiatry. 2016;29(1):13–7. 26575295 10.1097/YCO.0000000000000217

[94] Visseren FLJ, Mach F, Smulders YM, et al. 2021 ESC guidelines on cardiovascular disease prevention in clinical practice. Eur Heart J. 2021;42(34):3227–337. 34458905 10.1093/eurheartj/ehab484

[95] Peng P, Wang Y, Li Z et al. A network analysis of the long-term quality of life and mental distress of COVID-19 survivors 1 year after hospital discharge. Front Public Health. 2023, 11. 10.3389/fpubh.2023.1223429 PMC10416228 37575111

[96] Jafri MR, Zaheer A, Fatima S, Saleem T, Sohail A. Mental health status of COVID-19 survivors: a cross sectional study. Virol J. 2022, 19(1). 10.1186/s12985-021-01729-3 PMC8733909 34991632

[97] Kronish IM, Shechter A. COVID-19 and the amplification of cardiovascular risk by psychological distress. Nat Cardiovasc Res. 2022;1(11):968–70. 37138789 10.1038/s44161-022-00153-2 PMC10153572

[98] Komiyama M, Hasegawa K. Coronavirus disease 2019: psychological stress and cardiovascular diseases. Eur Cardiol. 2021;16:e33. 34603513 10.15420/ecr.2021.10 PMC8477172

[99] Gonjilashvili A, Tatishvili S. The interplay between Sars-Cov-2 infection related cardiovascular diseases and depression. Common mechanisms, shared symptoms. Am Heart J Plus. 2024;38:100364. 38510743 10.1016/j.ahjo.2024.100364 PMC10945907

[100] Mattioli AV, Coppi F, Nasi M, Gallina S. Stress and cardiovascular risk burden after the pandemic: current status and future prospects. Expert Rev Cardiovasc Ther. 2022;20(7):507–13. 35727895 10.1080/14779072.2022.2092097

